# Recurrent Epistaxis as a Rare Presentation of Extramedullary Multiple Myeloma

**DOI:** 10.7759/cureus.114795

**Published:** 2026-08-19

**Authors:** Vinay Kumar, Vinita Paswan, Prachi Kumar, Rahul Bhargava

**Affiliations:** 1 Otolaryngology-Head and Neck Surgery, Mahamaya Rajkiya Allopathic Medical College, Akbarpur, IND; 2 Pathology and Laboratory Medicine, All India Institute of Medical Sciences (AIIMS), Raebareli, IND; 3 Histopathology, Yashoda Medicity, Ghaziabad, IND; 4 Ophthalmology, GS Medical College & Hospital, Pilkhuwa, IND

**Keywords:** epistaxis, extramedullary disease, lytic bone lesions, multiple myeloma, plasma cell neoplasm, sinonasal mass

## Abstract

Extramedullary multiple myeloma is characterized by clonal plasma cell proliferation outside the bone marrow and is associated with an adverse prognosis, particularly when accompanied by high-risk cytogenetic abnormalities. Sinonasal involvement is rare and may present with recurrent epistaxis or unilateral nasal obstruction, creating diagnostic overlap with inflammatory, vascular, lymphoid, and epithelial sinonasal lesions. We report a 54-year-old man with an eight-month history of progressive right-sided nasal obstruction and intermittent epistaxis that became increasingly frequent and refractory to nasal packing. Magnetic resonance imaging showed a right sinonasal mass involving the nasal cavity and maxillary sinus, with septal deviation and no radiological evidence of orbital floor or nasal septal erosion. Endoscopically guided biopsy demonstrated a dense plasma cell infiltrate with mature and atypical forms. Systemic evaluation revealed anemia, multiple lytic skull and humeral lesions, 32% bone marrow plasma cell infiltration, an IgG monoclonal protein with lambda light-chain restriction, and high-risk cytogenetic abnormalities including CKS1B 1q gain, deletion 17p, and deletion 13q. These findings established a diagnosis of high-risk symptomatic multiple myeloma with extramedullary sinonasal involvement rather than a solitary extramedullary plasmacytoma. This case emphasizes that recurrent epistaxis associated with a unilateral sinonasal mass warrants early biopsy and systemic evaluation, particularly when clinical or laboratory features suggest an underlying plasma cell neoplasm.

## Introduction

Multiple myeloma is diagnosed when clonal bone marrow plasma cells account for at least 10% of marrow cellularity, or when there is a biopsy-proven bony or extramedullary plasmacytoma, together with one or more myeloma-defining events such as hypercalcemia, renal impairment, anemia, lytic bone disease, or recognized biomarkers of malignancy [1]. Extramedullary multiple myeloma refers to clonal plasma cell proliferation outside the bone marrow, either as soft-tissue masses arising by direct extension from skeletal lesions after cortical breach or through hematogenous spread to distant sites. Although its pathogenesis remains incompletely understood, extramedullary disease is frequently associated with high-risk cytogenetic features and adverse outcomes [2,3].

The clinical presentation of extramedullary disease is heterogeneous and depends largely on the site involved. Head and neck soft-tissue involvement is uncommon and may present with unilateral nasal obstruction, epistaxis, facial swelling, pain, or rhinorrhea [4,5]. Diagnosis requires tissue confirmation by biopsy or fine-needle aspiration with immunohistochemistry, along with a systemic myeloma workup that includes bone marrow examination. We describe a rare non-osseous sinonasal presentation of extramedullary multiple myeloma in a 54-year-old man who presented with progressive right-sided nasal obstruction and recurrent epistaxis.

## Case presentation

**Figure 1:**
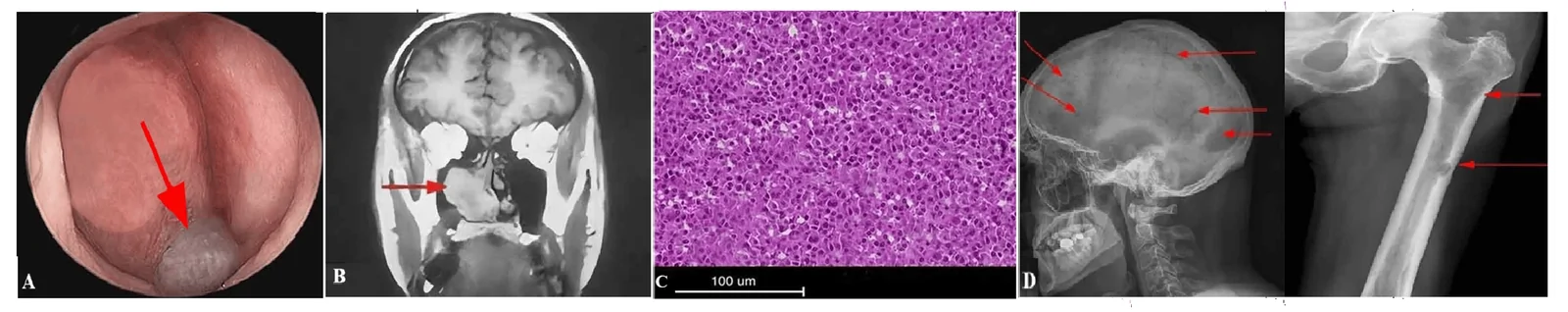
Rhinoscopy, radiological, and histopathological findings demonstrating sinonasal involvement and skeletal lytic lesions. (A) Anterior rhinoscopy revealed an obstructive right nasal mass (red arrow). (B) Contrast-enhanced T1-weighted coronal magnetic resonance image showing a mass involving the right maxillary sinus and nasal cavity, indicated by the red arrow. (C) Haematoxylin and eosin-stained nasal biopsy specimen at ×400 magnification demonstrating a dense plasma cell infiltrate composed of mature and atypical forms. (D) Plain radiographs of the skull and humerus demonstrating multiple lytic lesions, indicated by red arrows.

A man in his 50s presented with recurrent epistaxis and an eight-month history of progressive right-sided nasal obstruction. He had required anterior nasal packing for two previous bleeding episodes; however, during the 10 days before admission, the epistaxis became more frequent and less responsive to conservative measures. He also reported snoring, weight loss, and reduced exercise tolerance. There was no history of local trauma. Anterior rhinoscopy (red arrow) revealed an obstructive right nasal mass (Figure 1A). Magnetic resonance imaging demonstrated a moderately enhancing mass involving the right maxillary sinus and nasal cavity (red arrow), without prominent flow voids, tumour blush, or erosion of the orbital floor or nasal septum (Figure 1B). Based on the site and imaging appearance, provisional considerations included inflammatory sinonasal disease, primary sinonasal malignancy, lymphoma, plasmacytoma, and vascular lesions; however, the absence of marked vascularity made a highly vascular tumour less likely.

Endoscopically guided incisional biopsy was performed, and the specimen was submitted for histopathological examination. Anterior nasal packing was applied for haemostasis. Microscopy showed a dense plasma cell infiltrate composed of mature and atypical forms (Figure 1C). Plain radiographs of the skull and humerus demonstrated multiple lytic lesions consistent with myeloma-defining bone disease (Figure 1D). Monoclonality was supported by serum immunofixation findings; additional tissue-based light-chain restriction studies were not available in the submitted material. Table 1 shows the baseline laboratory values and their reference ranges.

**Table 1:** Baseline laboratory investigations with patient values and reference ranges.

Parameters	Patient values	Reference range
Haemoglobin	7.6 g/dL	Male: 13.5–17.5 g/dL; female: 12.0–15.5 g/dL
White blood cell count	4,500/mm³	4,500–11,000/mm³
Platelet count	109,000/µL	150,000–400,000/µL
Sodium	139 mEq/L	136–146 mEq/L
Potassium	4.1 mEq/L	3.5–5.0 mEq/L
Calcium	9.1 mg/dL	8.4–10.2 mg/dL
Phosphorus	4.1 mg/dL	3.0–4.5 mg/dL
Total protein	10.2 g/dL	6.0–7.8 g/dL
Albumin	5.2 g/dL	3.5–5.5 g/dL
Globulin	5.0 g/dL	2.3–3.5 g/dL
Creatinine	1.18 mg/dL	0.6–1.2 mg/dL
Blood urea nitrogen	25 mg/dL	7–18 mg/dL
Prothrombin time	24.1 seconds	11–13.5 seconds
International normalized ratio	1.75	0.8–1.1
Serum-free kappa light chain	0.34 mg/dL	0.33–1.94 mg/dL
Serum-free lambda light chain	5.6 mg/dL	0.57–2.63 mg/dL
Kappa/lambda ratio	0.06	0.26–1.65

**Figure 2:**
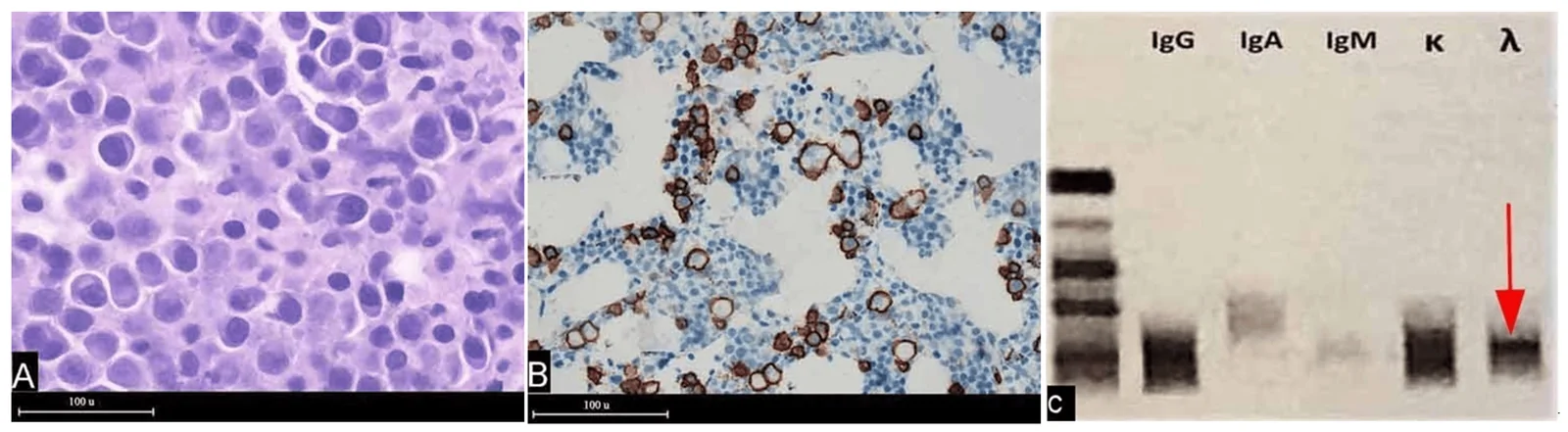
Bone marrow, immunohistochemical, and immunofixation findings. (A) Haematoxylin and eosin-stained bone marrow trephine biopsy at X 400 magnification showing significant plasma cell infiltration, including mature forms with eccentric nuclei and a perinuclear hola, as well as plasmablasts. (B) High-power immunostaining with CD138 stain at ×400 magnification confirming the plasma cell lineage in the bone marrow. (C) Immunofixation electrophoresis demonstrating an IgG monoclonal protein with lambda light-chain restriction, indicated by the red arrow.

Bone marrow trephine biopsy from the posterior superior iliac spine showed 32% plasma cell infiltration, including mature forms with eccentric nuclei and a perinuclear halo, binucleate and trinucleate plasma cells, and plasmablasts (Figure 2A). Immunohistochemistry confirmed the plasma cell lineage with CD138 positivity (Figure 2B). Serum protein electrophoresis showed a monoclonal spike in the gamma region, and immunofixation identified an IgG monoclonal protein with lambda light-chain restriction (Figure 2C). Cytogenetic analysis demonstrated CKS1B 1q gain, deletion 17p, and deletion 13q, consistent with high-risk cytogenetic disease. Collectively, the sinonasal plasma cell infiltrate, IgG lambda monoclonal protein, marrow plasmacytosis, anemia, multifocal lytic bone lesions, and high-risk cytogenetic abnormalities supported a diagnosis of high-risk symptomatic multiple myeloma with extramedullary sinonasal involvement. Table 2 shows the clinical timeline, summary of symptom onset, diagnostic evaluation, systemic assessment, cytogenetic findings, final diagnosis, and initial management plan. 

**Table 2:** Clinical timeline, summary of symptom onset, diagnostic evaluation, systemic assessment, cytogenetic findings, final diagnosis, and initial management plan.

Time point	Clinical events and findings
Approximately eight months before admission	Progressive right-sided nasal obstruction with intermittent episodes of epistaxis began.
Before current admission	Two prior episodes of epistaxis were controlled with anterior nasal packing.
10 days before admission	Epistaxis increased in frequency and became less responsive to conservative measures.
At presentation	The patient reported recurrent epistaxis, right-sided nasal obstruction, snoring, weight loss, and reduced exercise tolerance; anterior rhinoscopy identified an obstructive right nasal mass.
Initial imaging	Contrast-enhanced T1-weighted coronal MRI showed a mass involving the right maxillary sinus and nasal cavity, with leftward septal deviation and no radiological evidence of bony erosion.
Tissue diagnosis	Endoscopically guided incisional biopsy showed a dense plasma cell infiltrate composed of mature and atypical forms.
Systemic evaluation	Laboratory assessment showed anaemia, monoclonal gamma-region spike, IgG lambda restriction on immunofixation, serum-free kappa 9.4 mg/dL, serum-free lambda 5.6 mg/dL, and a kappa/lambda ratio of 1.68.
Skeletal and marrow assessment	Plain radiographs demonstrated multiple lytic skull and humeral lesions. Bone marrow trephine biopsy showed 32% plasma cell infiltration with CD138 positivity.
Cytogenetic assessment	Cytogenetic analysis demonstrated CKS1B 1q gain, deletion 17p, and deletion 13q, supporting high-risk cytogenetic disease.
Final diagnosis and management plan	High-risk symptomatic multiple myeloma with extramedullary sinonasal involvement was diagnosed. The patient was referred to onco-haematology for bortezomib-based induction therapy, with consideration of autologous stem-cell transplantation.

Differential diagnosis

The main diagnostic considerations for a unilateral sinonasal mass with recurrent epistaxis included inflammatory or vascular sinonasal lesions, primary sinonasal malignancy, lymphoma, extramedullary plasmacytoma, and systemic multiple myeloma with extramedullary involvement. The biopsy-proven plasma cell infiltrate established a plasma cell neoplasm, while anemia, multifocal lytic bone lesions, 32% marrow plasma cell infiltration, IgG lambda monoclonal protein, and high-risk cytogenetic abnormalities distinguished systemic multiple myeloma from solitary extramedullary plasmacytoma.

Treatment

Active bleeding was controlled with anterior nasal packing. After multidisciplinary review, neither surgical intervention nor embolization was pursued. The patient was referred to onco-hematology for bortezomib-based induction therapy and evaluation for autologous stem-cell transplantation.

Outcome and follow-up

At the time of manuscript preparation, the patient had been referred for systemic anti-myeloma therapy and transplant eligibility assessment. Detailed post-treatment response data and long-term follow-up were not yet available.

## Discussion

Extramedullary multiple myeloma involving the nasal cavity and paranasal sinuses is rare, and presentation with recurrent epistaxis may delay recognition of the underlying plasma cell disorder. Reported sites of extramedullary involvement include soft tissue adjacent to bone, lymph nodes, liver, spleen, kidneys, lungs, skin, central nervous system, testes, breast, and the gastrointestinal tract; sinonasal involvement remains uncommon [6]. Classic manifestations of multiple myeloma include bone pain, anemia, pathological fractures, renal dysfunction, and recurrent infections. In extramedullary disease, localized symptoms may precede or overshadow these typical features, contributing to diagnostic delay [7].

Extramedullary disease is reported in approximately 1-4% of newly diagnosed cases and up to 10% of relapsed cases, most often in advanced disease. Proposed mechanisms include altered adhesion molecule expression, dysregulated signaling pathways, and disrupted interactions between plasma cells and the bone marrow microenvironment [8,9]. In this patient, CKS1B 1q gain, deletion 17p, and deletion 13q indicated a biologically high-risk profile, reinforcing the need for prompt hematology referral and systemic therapy rather than local treatment alone.

From an oncological perspective, the sinonasal lesion represented systemic high-risk multiple myeloma with extramedullary involvement rather than an isolated local plasma cell tumour. The combination of anemia, multifocal lytic bone disease, marrow plasmacytosis, monoclonal IgG lambda protein, and adverse cytogenetics, including CKS1B 1q gain and deletion 17p, indicated aggressive disease biology and supported systemic risk-adapted anti-myeloma therapy rather than local treatment alone. Complete staging with marrow assessment, monoclonal protein studies, skeletal imaging, and cytogenetic evaluation was therefore essential to guide management, including bortezomib-based induction and assessment for autologous stem-cell transplantation.

Sinonasal involvement by plasma cell neoplasms requires a broad differential diagnosis. Relevant considerations include extramedullary plasmacytoma, systemic multiple myeloma with extramedullary involvement, lymphoma, primary head and neck malignancies such as squamous cell carcinoma, and selected benign inflammatory or vascular lesions. Accurate diagnosis depends on cross-sectional imaging, tissue biopsy, immunohistochemistry, and systemic evaluation. Younis et al. reported a solitary plasmacytoma presenting with unilateral epistaxis; in contrast, this case showed systemic myeloma features, including marrow involvement, anemia, monoclonal protein, multifocal lytic bone disease, and high-risk cytogenetic abnormalities [10]. 

Before biopsy of a bleeding sinonasal mass, vascularity should be assessed carefully on contrast imaging. Marked enhancement, flow voids, tumour blush, or extension patterns suggestive of lesions such as juvenile nasopharyngeal angiofibroma, haemangioma, or paraganglioma should prompt avoidance or deferral of biopsy because of haemorrhage risk, with angiography or embolization considered when appropriate. In this case, imaging did not show marked vascularity, and the patient's systemic features, including anaemia, hyperproteinaemia, lytic bone lesions, marrow plasmacytosis, and monoclonal IgG lambda protein, favoured plasma cell neoplasm; therefore, endoscopically guided biopsy was performed to confirm the diagnosis.

## Conclusions

Non-osseous extramedullary multiple myeloma may rarely present as a sinonasal mass with recurrent epistaxis. Because this presentation can mimic inflammatory, vascular, or malignant sinonasal disease, clinicians should consider a plasma cell neoplasm when persistent unilateral nasal symptoms are accompanied by systemic features or abnormal imaging findings. Early biopsy, systemic evaluation, and hematology referral are essential to distinguish solitary extramedullary plasmacytoma from systemic multiple myeloma and to guide timely treatment.
